# How to optimise public health interventions: a scoping review of guidance from optimisation process frameworks

**DOI:** 10.1186/s12889-020-09950-5

**Published:** 2020-12-02

**Authors:** Sam McCrabb, Kaitlin Mooney, Benjamin Elton, Alice Grady, Sze Lin Yoong, Luke Wolfenden

**Affiliations:** 1 School of Medicine and Public Health, Faculty of Health and Medicine, University of Newcastle, 1 University Drive, Callaghan, NSW 2308 Australia; 2 Hunter New England Population Health, Hunter New England Local Health District, Wallsend, NSW 2287 Australia

**Keywords:** Optimisation, Scoping review, Framework, Public health, Intervention, Implementation, Intervention development

## Abstract

**Background:**

Optimisation processes have the potential to rapidly improve the impact of health interventions. Optimisation can be defined as *a deliberate, iterative and data-driven process to improve a health intervention and/or its implementation to meet stakeholder-defined public health impacts within resource constraints.* This study aimed to identify frameworks used to optimise the impact of health interventions and/or their implementation, and characterise the key concepts, steps or processes of identified frameworks.

**Methods:**

A scoping review of MEDLINE, CINAL, PsycINFO, and ProQuest Nursing & Allied Health Source databases was undertaken. Two reviewers independently coded the key concepts, steps or processes involved in each frameworks, and identified if it was a framework aimed to optimise interventions or their implementation. Two review authors then identified the common steps across included frameworks.

**Results:**

Twenty optimisation frameworks were identified. Eight frameworks were for optimising interventions, 11 for optimising implementation and one covered both intervention and implementation optimisation. The mean number of steps within the frameworks was six (range 3–9). Almost half (*n* = 8) could be classified as both linear and cyclic frameworks, indicating that some steps may occur multiple times in a single framework. Two meta-frameworks are proposed, one for intervention optimisation and one for implementation strategy optimisation. Steps for intervention optimisation are: Problem identification; Preparation; Theoretical/Literature base; Pilot/Feasibility testing; Optimisation; Evaluation; and Long-term implementation. Steps for implementation strategy optimisation are: Problem identification; Collaborate; Plan/design; Pilot; Do/change; Study/evaluate/check; Act; Sustain/endure; and Disseminate/extend.

**Conclusions:**

This review provides a useful summary of the common steps followed to optimise a public health intervention or its implementation according to established frameworks. Further opportunities to study and/or validate such frameworks and their impact on improving outcomes exist.

**Supplementary Information:**

The online version contains supplementary material available at 10.1186/s12889-020-09950-5.

## Background

Considerable public funding is invested globally in the development and delivery of interventions to improve patient and public health. The benefits of such investments are increasingly being scrutinised [1]. Often, health interventions that are examined in research trials are found to have no beneficial impact, or achieve only modest improvements in health outcomes even when tested under ideal research conditions [2, 3]. A further constraint to the impact of government investment in health initiatives is the challenge of implementation [4]. That is, even when efficacious interventions are identified, their effects typically attenuate when delivered in more real world contexts – due, in part, to poor implementation [5, 6]. Identifying both interventions that are effective in the ‘real world’ and effective strategies to implement them is required to maximise the translation of research into effective policy and practice.

Rarely are improvements in health care characterised by scientific ‘break through’ discoveries that yield immediate and large improvements in patient or population health outcomes. Rather, improvements tend to occur incrementally, as new knowledge generated through scientific research regarding the determinants and treatment of disease accumulates [7, 8]. A number of factors impede the efficient accumulation and application of evidence to improve health care, including differences in research design features, measures and contexts that make comparison and synthesis of study findings problematic [9]. Such factors lead to considerable research waste, and slow scientific progression and health care improvement.

Optimisation processes have the potential to transform health care through accelerating incremental improvements in the impact of interventions via the co-ordinated testing of interventions using comparable methods and contexts. Optimisation is inherent in quality improvement processes applied in the manufacturing, information technology and engineering sectors to improve the performance of products, and has been applied in medicine to improve the quality of health care [10, 11]. For example, the use of ‘implementation laboratories’ have industrialised the research production process to optimise strategies such as audit and feedback, to improve professional practice and the implementation of therapeutic interventions [12].

In the field of public health, optimisation has been defined as a *“deliberate, iterative and data-driven process to improve a health intervention and/or its implementation to meet stakeholder-defined public health impacts within resource constraints”* [13]. While it is a relatively new concept in public health there are a number of examples where optimisation processes have been employed. The Questions about Quitting [14] trial used a formal optimisation framework (Multiphase Optimisation Strategy; ‘MOST’) to improve the impact of a smoking cessation intervention. Specifically, through ongoing experimentation the study aimed to identify the most effective and efficient combination of intervention components [15]. Optimisation processes have also been applied to improve public health programme implementation. For example, across a series of randomised controlled trials aiming to increase school implementation of food availability policies, modifications to a strategy to improve the implementation of a school nutrition policy led to an almost halving of the incremental cost-effectiveness ratio [16].

Despite a number of examples of optimisation in the fields of medicine and public health, there has been considerable differences in approaches and methods employed to optimise impact [2, 11, 17]. Frameworks may provide a useful tool for researchers and practitioners to guide the application of optimisation methods and processes in public health and medicine, or assist in determining the point at which once optimisation has been achieved. In 2014, Levati et al. [2] conducted a scoping review of strategies used to optimise the effectiveness of behavioural interventions before being evaluated in a full scale randomised controlled trial. They identified frameworks such as MOST [18], the Medical Research Council (MRC) framework from 2000 [19] and 2008 [20], Process Modelling in Implementation Research (PRIME) [21], and Normalisation Process Theory (NPT) [22] as commonly used to guide processes to enhance the effects of interventions through optimisation. However, the review examined frameworks used in the optimisation of interventions and their components during intervention development, rather than strategies to improve strategies to facilitate their implementation. Both effective interventions, and implementation strategies are required to maximise the public health impact of evidence based initiatives. Reed and colleagues (2018) [23] conducted a literature review to compare the authors’ framework titled ‘SHIFT-Evidence’ to 10 popular implementation and improvement frameworks. However, the frameworks examined were purposely selected, rather than systematically identified. Additionally, none of these reviews synthesised the characteristics of steps in included frameworks.

Given the potential and interest in the application of optimisation for public health improvement [13], the systematic identification of frameworks relevant to optimisation in public health and medicine, and the characterisation of commonly recommended processes inherent in these frameworks may represent an important resource to guide future optimisation research and practice. As such we sought to conduct a scoping review to:

1. identify frameworks to optimise the impact of health care and public health initiatives (interventions and/or implementation strategies); and
2. characterise the key concepts, steps or processes of identified frameworks.

## Methods

The scoping review followed the recommended methods described by the Johanna Briggs Institute [24].

### Study inclusion and exclusion criteria

Publications were included if:

1. They report a framework describing a process of optimisation defined as a *“deliberate, iterative and data-driven process to improve a health intervention and/or its implementation to meet stakeholder-defined public health impacts within resource constraints”* [13]*.*
2. They described a process framework for improving health outcomes, intervention effectiveness or implementation. Adapting the Moullin et al. [25] definition, we defined a framework as *any graphical or narrative representation of the key factors, concepts, or variables to explain a deliberative process to improve the impact of a health service or intervention.* A process framework was defined as a framework which aims to provide direct steps for guidance, i.e. specify steps/stages/phases to describe or guide the processes of optimisation [26, 27]. We excluded determinant frameworks which describe factors related to the concept of optimisation i.e. specific factors such as barriers and facilitators which may influence or explain the outcomes of optimisation [26, 27].
3. Describe a framework applicable to public health, medical, or health services. Specifically, included frameworks must have been either a) applied in a public health-based, health or medical setting (e.g. hospital, doctors surgery, clinical or community health, schools etc.), or b) clearly stated that the frameworks can be applied to public health-based, health or medical interventions. Frameworks which developed specifically for use in other sectors such as manufacturing, information technology or agriculture were excluded.
4. They describe a framework that explicitly seeks to be used to improve the effectiveness of interventions (i.e. patient/participant acceptability, cost-effectiveness, intervention effectiveness) and/or the effectiveness of strategies to implement an intervention (e.g. fidelity of delivery, costs, feasibility etc.).

Non-English language studies were excluded. Based on the publication dates of included studies in a review by Levati et al. (2015) [2] we restricted the search to studies published in the last 15 years (2003–2018). We then updated the search January 2019.

### Search strategy

Given limited research in the area, an initial search using keywords and subject headings was conducted to develop search terms sensitive enough to capture all potentially eligible articles. Following a review of the initial search results, a second search with more applicable keywords was conducted in January 2019. Search terms for relevant frameworks were developed based on terminology used by Levati et al. [2] (optimisation), Kaplan et al. [28] (quality improvement; QI), Gardner et al. [29] (continuous quality improvement; CQI), Kaplan et al. [28] and Gardner et al. [29] (health context), and Mouillin et al. [25](frameworks). Supplementary File 1 contains a full list of the search process for both searches. MEDLINE, CINAL, PsycINFO, and ProQuest Nursing & Allied Health Source databases were searched to identify potentially relevant articles. All studies were assessed for eligibility. When an identified study cited an existing optimisation framework, but was not the original developer of the framework, the original framework was traced back using Google Scholar so that it could be assessed against the inclusion/exclusion criteria. Additionally, reference lists of relevant reviews [2, 23] and the reference lists of all included studies were also screened for relevant frameworks.

### Study selection

Pairs of unblinded reviewers (SM^C^, BE, AB, ED, MM – see acknowledgements) independently screened titles and abstracts. Screening of studies was conducted using Covidence systematic review software [30]. The full texts of manuscripts were obtained for all potentially eligible studies for further examination. Pairs of review authors (SM^c^, BE, LKC, KM) first screened 5% of potentially relevant studies together to ensure agreement prior to completing full text review, again unblinded and independently. For all full text manuscripts, information regarding the primary reason for exclusion was recorded. Uncertainties between reviewers regarding study eligibility were resolved by consensus or consultation with a third reviewer (SM^c^, BE, LKC, KM). Searches of existing frameworks were screened by pairs of reviewers with uncertainties resolved by consensus (SMc, BE, KM).

### Data extraction and management

Pairs of review authors (SM^c^ and AG or KM), independently extracted information from the included studies. This information was recorded in a bespoke data-extraction form that was piloted before initiation of the review. The following information was extracted (Table 1):

1. Study characteristics: author, year, country.
2. Framework characteristics: name; description of the steps for optimisation; the number of steps; whether a figure is available (yes/no); whether the framework included any descriptive guidance (yes/no); whether the framework format is linear (follows a step by step sequential process), cyclic (steps could be repeated or you could return to an earlier step in the framework), both or other; whether there is any description of an optimisation endpoint (i.e. when optimisation is achieved); whether the framework was intended to be used for optimising an intervention (e.g. intervention effectiveness) and/or its implementation (e.g. intervention adoption); whether the framework was modified from another framework (yes/no), and if so, the name (and reference) for the original framework.
3. Outcomes the framework was designed to improve were also extracted and classified into categories defined by Proctol et al. [31] including implementation (acceptability, adoption, appropriateness, costs, feasibility, fidelity, penetration, and sustainability), service (efficiency, safety, effectiveness, equity, patient-centeredness, and timeliness), and patient (satisfaction, function and symptomatology) level outcomes. An ‘other’ category was also established for outcomes that could not be otherwise categorised.

**Table 1:** Data extracted from each included study

Classifications of data extracted	Data extracted	Data extracted- sub-categories
Study characteristics	Author	–
	Year of publication	–
	Country	–
Framework characteristics	Framework name	–
	Description of the steps for optimisation	–
	Number of steps	–
	Figure available	Yes/No
	Framework format	Linear, cyclic, both linear and cyclic
	Description of optimisation endpoint	–
	What the framework optimises	Intervention, implementation, both
	Was the framework modified from another framework	Yes (and reference)/No
Outcomes the frameworks were designed to improve	Implementation	Acceptability, adoption, appropriateness, costs, feasibility, fidelity, penetration, and sustainability
	Service	Efficiency, safety, effectiveness, equity, patient-centeredness, and timeliness
	Patient	Satisfaction, function and symptomatology
	Other	–

### Data synthesis

For aim 1, data extracted was synthesised according to whether the framework was designed to optimise an intervention and/or its implementation. To describe the framework characteristics and outcomes optimised, descriptive statistics were collated in tables and presented as numbers and percentages for categorical variables and means (standard deviation) or median (interquartile range) for continuous variables, depending on distribution of the data. All extracted data were entered into an Excel 2013 spreadsheet for analysis.

For aim 2, narrative synthesis of the key themes of steps involved in each framework was conducted by two authors (SMc & LW) and are reported below.

Following the methods used by Escoffery et al. [32] in their scoping review of adaptation frameworks, two review authors were responsible for the mapping and collating of the steps included in identified frameworks. Initially, one review author (SMc) independently extracted information regarding the details of steps of included frameworks into Excel. One review author (SMc) then reviewed and identified common elements of each framework and noted shared steps for synthesis. Separately, a second review author (LW) reviewed included frameworks and details of the steps involved. Together, both review authors (SMc and LW) then mapped the common steps creating two synthesised meta-frameworks, one for intervention optimisation and one for implementation optimisation. A step was only included in the meta-framework if it was mentioned by at least two of the included frameworks. In the instance where only one framework was deemed to mention a potentially important step, this was noted down for discussion. Sub-steps were included if frameworks had a similar method, but spilt the process up over multiple steps.

## Results

**Fig. 1:**
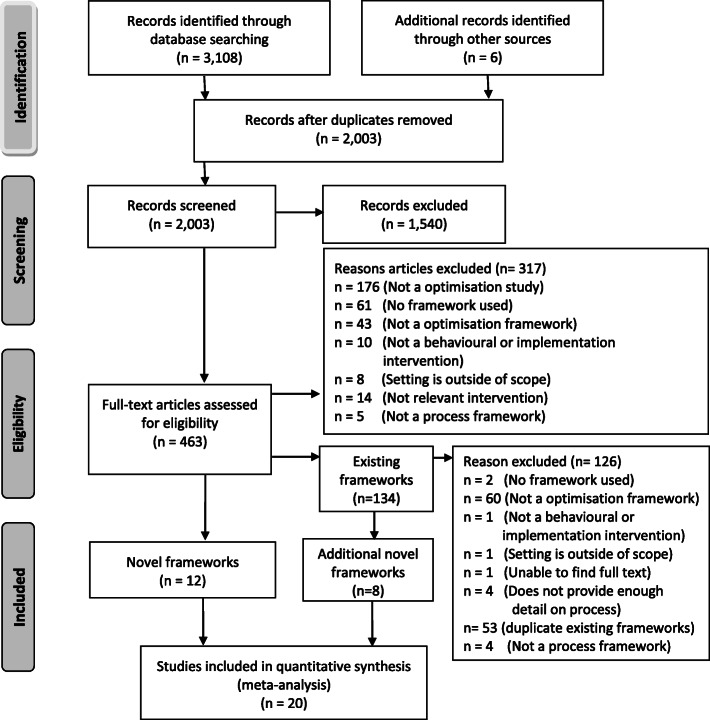
Flow diagram depicting the movement of studies through the review

Of the 2003 citations screened, 463 were identified as potentially eligible and full text manuscripts were obtained for further eligibility assessment (Fig. 1). Of these, 20 frameworks were included. Characteristics of included frameworks are summarised in Supplementary File 2. The primary reason for exclusion following full text screening were: not an optimisation study; no framework was used; or the framework used was not an optimisation framework.

A summary of the characteristics of included frameworks can be found in Table 2. Included articles were published from 1996 to 2019. The date range lays outside our search date range as back tracing existing frameworks through Google Scholar and searching key systematic review identified additional frameworks. The majority of frameworks were either cyclic (*n* = 8) or included both linear and cyclic processes (n = 8). Approximately one third (*n* = 6) of the frameworks specified an endpoint, that is, a point in the framework when optimisation was achieved. Four of these six frameworks included the end point of reaching a specific step or milestone in the framework [33–36]. One of these six determined the end-point to be reached once the most effective intervention which could be achieved had been developed [37]. One stated optimisation to be achieved once a predefined question has been sufficiently answered [38]. Almost one third (*n* = 6) of the frameworks [20, 34, 39–42] were identified as being modified, or incorporating components from a prior framework.

**Table 2:** Summary of characteristics of included frameworks

Characteristic	Total = 20 n (%)
Number of steps, mean (SD)	5.95 (1.99)
Figure available for framework (yes)	14 (70%)
Explicit guidance for each step available (yes)	19 (95%)
Framework format	
Linear	4 (20%)
Cyclic	8 (40%)
Both linear and cyclic	8 (40%)
Endpoint specified (yes)	6 (30%)
Optimisation focus	
Optimising Intervention	8 (40%)
Optimising Implementation	11 (55%)
Optimising both Intervention and Implementation	1 (5%)
Modified from other frameworks (yes)	6 (30%)

Across frameworks the average number of steps or phases in an optimisation process was six (range 3–9) (Table 2). Eight of the frameworks were developed to optimise an intervention, six to optimise implementation of an intervention, and one framework explicitly intended to be applied to both intervention and implementation optimisation [39]. Data from this framework is presented twice as is addressed both intervention and implementation processes.

The outcomes which frameworks explicitly stated they were intended to optimise (or which had been reportedly applied to optimise) are presented in Table 3. No one framework explicitly intended or had been applied to optimise all of the 17 implementation, service or client outcomes according to Proctor [31]. The most common outcomes to be optimised were: effectiveness, efficiency and cost. The three most infrequently optimised outcomes were patient-centredness, symptomatology and penetration. Four frameworks did not specify the outcomes they improved as these were set by the improvement team as part of the optimisation framework [39, 41], or were related to the concept aiming to be improved [43, 44].

**Table 3:** Included review outcomes optimised by frameworks, mapped to 17 outcomes according to Proctor et al. [31]

	Outcome optimised by framework	Total ***n*** = 20 n (%)
Implementation	Acceptability: Satisfaction with various aspects of the innovation (e.g. content, complexity, comfort, delivery, and credibility)	4 (20%)
	Adoption: Uptake; utilisation; initial implementation; intention to try	2 (10%)
	Appropriateness: Perceived fit; relevance; compatibility; suitability; usefulness; practicability	2 (10%)
	Cost: Marginal cost; cost-effectiveness; cost-benefit	7 (35%)
	Feasibility: Actual fit or utility; suitability for everyday use; practicability	4 (20%)
	Fidelity: Delivered as intended; adherence; integrity; quality of program delivery	4 (20%)
	Penetration: Level of institutionalisation, spread, service access	1 (5%)
	Sustainability: Maintenance; continuation; durability; incorporation; integration; institutionalisation; sustained use; routinisation.	4 (20%)
Service	Efficiency: Avoiding waste, including waste of equipment, supplies, ideas, and energy.	10 (50%)
	Safety: Avoiding harm to patients from the care that is intended to help them.	2 (10%)
	Effectiveness: A measure of how well a program/policy performs in a real world setting where variables cannot be controlled.	11 (55%)
	Equity: Providing care that does not vary in quality because of personal characteristics such as gender, ethnicity, geographic location, and socioeconomic status.	2 (10%)
	Patient-centredness: Providing care that is respectful of and responsive to individual patient preferences, needs, and values and ensuring that patient values guide all clinical decisions.	0 (0%)
	Timeliness: A measure of how often waits and harmful delays occur for both those who receive and those who give care.	6 (30%)
Patient	Patient' Satisfaction: extent to which a client is content with the service which they received.	5 (25%)
	Function: A measure of participant’s functional status e.g. their ability to perform normal daily activities required to meet basic needs, fulfil usual roles, and maintain health and well-being.	3 (15%)
	Symptomatology: the set of symptoms characteristic of a medical condition or exhibited by a patient.	1 (5%)

### Characterisation of key concepts, steps or processes of identified frameworks

Synthesis of the key steps and processes identified distinct differences between those frameworks intended to optimise an intervention, and those intended to optimise the implementation of an intervention. As such, we synthesised each as two separate meta-frameworks.

#### Meta-frameworks for intervention optimisation

**Fig. 2:**
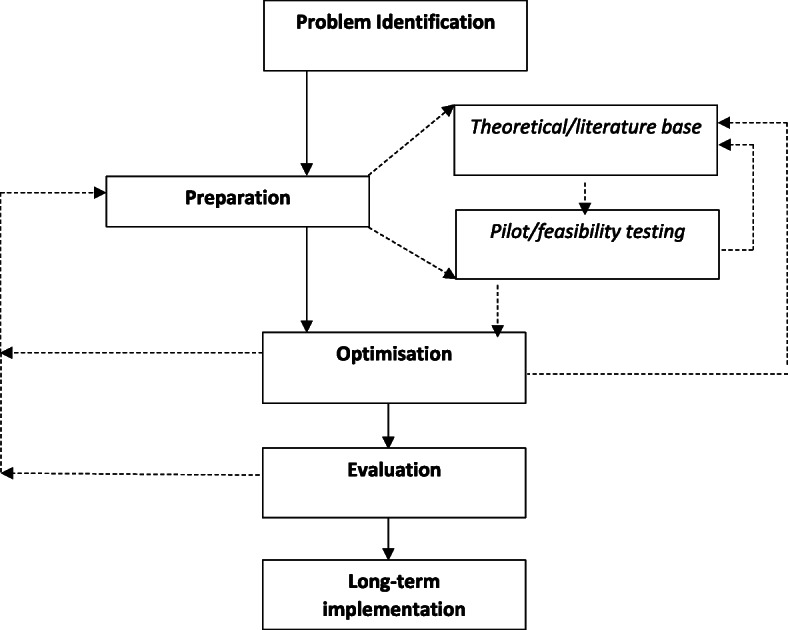
Meta-framework to optimise interventions *Italics* identifies sub-steps in this framework. Dotted lines indicates paths that interventions may take when following the framework. Not all intervention will return back to earlier steps, or they may return back to different steps depending on their progress through the framework

Among the eight frameworks used to optimise interventions, seven conceptual steps were identified. (Figure 2, examples from included frameworks synthesised are available in Supplementary File 3).

The first step was ‘*Problem identification’* where frameworks sought to identify the key parameters of the health issue on which an optimisation processes was to be applied to address. The frameworks typically would seek to describe the problem in terms of *“the clinical question or problem for which a behavioural treatment could provide a solution”* [36]. This is commonly identified by determining gaps in the research literature through reviews, or clinical identification of the ‘problem’ [40]. The second broad step, termed ‘*Preparation’*, outlined broadly how interventions were developed prior to investment in formal experimentation and evaluation. The preparation step may be split into two sub-steps (‘*Theoretical/literature base’* and *‘Pilot/feasibility testing’*). In the first, frameworks suggested using theoretical models, experience or scientific literature to develop the intervention and define the optimisation criteria. This may include formal specification of the likely components of the intervention to be optimised, through the use of programme theory or logic models [19, 20, 40]. Additionally, however, some frameworks suggested an additional step, in which proof of concept pilot or feasibility testing of the intervention were undertaken. Based on the findings of these pilot and feasibility studies interventions may cycle back to the ‘Theoretical/Literature base’ step for further development.

In the ‘*Optimisation’* step, frameworks suggest investigators undertake experiments to measure the performance of the intervention and/or its components against the defined optimisation criteria. The purpose of the optimisation step was to experiment and refine the intervention, and its programme theory or logic, to better understand intervention mechanisms and to improve its impact. Often this occurred through the use of multiple or iterative ‘mini experiments’. At this stage, the intervention could cycle back to the previous step (i.e. preparation or ‘theoretical/literature base’) if the ‘experimentation’ step was not successful. For example, Collins et al. [37] suggest the use of Sequential, Multiple Assignment, Randomised Trial (SMART) to test different components of an intervention. Using a SMART design, the researcher could randomise the sequence of factors of interest over time, using each randomisation stage as a decision point to address a specific question concerning two or more treatment options (e.g. is stress management training more effective than personalised normative feedback).

The penultimate step was ‘*Evaluation’* of the proposed optimised intervention. In this step, formal and often large confirmatory studies were undertaken to determine if the intervention was effective in achieving the desired impact usually defined in terms of the key optimisation criteria such as effectiveness or cost-effectiveness. If the intervention following the ‘Evaluation’ step was not found to be effective, frameworks may cycle back to the previous ‘Preparation’ step. An example of this step comes from Haji et al. [40] who state that if an evaluation fails to demonstrate expected outcomes, these results should be disseminated and the project investigators should identify an alternate theory, explore other intervention features of interest, and return to the evaluation step once the intervention has been revised through cycles of the preparation development and optimisation step as needed.

The final step commonly identified to optimise intervention frameworks was to determine the ‘*Long-term implementation’* of the intervention if deemed effective. This was conducted to determine if the intervention could maintain its effectiveness in an uncontrolled setting long-term. The MRC framework [19] provides guidance on this, stating that the long-term implementation step is conducted to determine real world effectiveness, outside of the confines of a research study. This step usually involves an observational study.

#### Meta-framework for implementation strategy optimisation

**Fig. 3:**
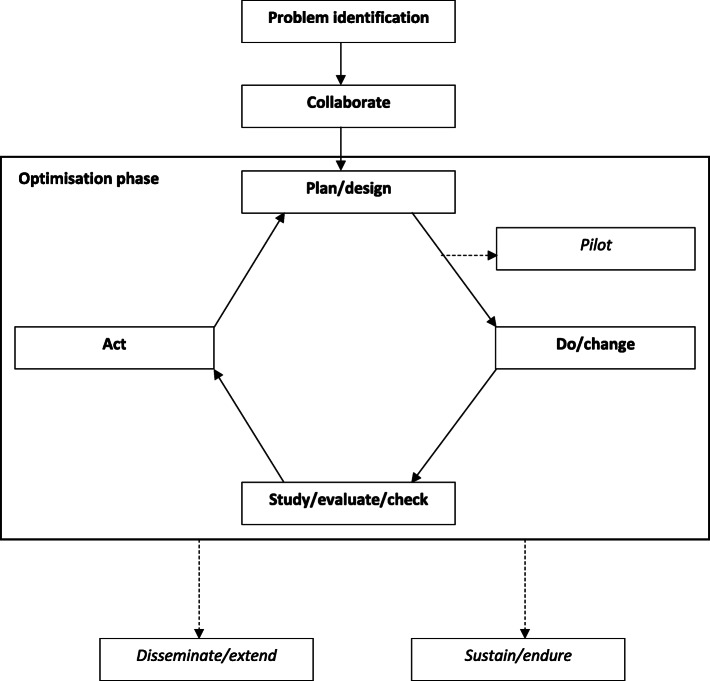
Meta-framework to optimise implementation *Italics* identifies sub-steps in this framework. Dotted lines indicates paths that interventions may take when following the framework. Not all intervention will return back to earlier steps, or they may return back to a different steps depending on their progress through the framework

The meta-framework for implementation strategy optimisation contained six steps (with an additional three sub-steps which were optional). The meta-framework was heavily influenced by quality improvement cycles which featured heavily in the included frameworks. Figure 3 shows a depiction of the synthesised framework and additional information can be found in Supplementary File 3.

Similar to the synthesised intervention framework, the first step in this diagram is ‘*Problem identification’* where key parameters of the health issue or implementation challenge on which an optimisation processes was to be applied to address was scoped and specified. For example, frameworks suggest the use of literature reviews and the collection of local data to appropriately characterise the issue. Specifically, the Institute for Clinical Systems Integration (ICSI) process by Mosser et al. [45] suggests population health surveys, individuals’ insights and diagnostic frequencies to inform topic choice. In the next step, ‘*Collaborate’,* existing optimisation frameworks suggests the establishment of key stakeholder teams and structures to lead, and inform the process of optimisation. Such groups should include all stakeholders who would be impacted by changes in the implementation of a targeted health intervention program or policy, for example, health managers, clinicians, patients or community representative, as well as researchers. McGonigal et al. [41] state that it is important to get the right people involved before deciding the direction an approach will take. Redick et al. [44] state team members should be from every discipline involved in the process and they should be chosen for their experience with the condition being studied/implemented rather than their job titles.

The following section of the framework is termed *‘Optimisation’* and describes broadly the process articulated in Plan-Do-Study-Act cycles, and demonstrates the considerable influence of Walter Shewhart and Edwards Deming (who taught the process for quality improvement) in the field of implementation [46]. Step three in the meta-framework ‘*Plan/design’* refers to the process of clarifying what modifications to behaviour, systems or process need to occur in order for the targeted intervention, policy or practice is to be implemented, in what context this is to occur, and what strategies should be employed to achieve this. Frameworks suggest this process could involve measurements of baseline performance to determine root causes and to enable changes in measurements [34]. Some frameworks also suggested an additional sub-step in this process, labelled ‘*Pilot’*, in which piloting of the suggested optimised implementation is trialled. Mosser et al. [45] outlined that their trial period was for 4 months, with data collected for the stakeholder group who met up upon completion of the trial to determine effectiveness of the proposed changes. If successful, the stakeholder group would approve the changes for greater implementation. This acts as an additional quality check, to ensure the measures are collecting the data needed to determine implementation effectiveness.

Following this, the ‘*Do/change’* step is where the implementation strategy is executed and implementation of the intervention, policy or practice occurs. For example, in the Routine Outcome Measurement (ROM) framework [43], this action is referred to as informing selected changes to practice. McKay et al. [43] state that at times this may require a staged approach. Redick refers to this step simply as ‘implement the developed plan’. [44]

‘*Study/evaluate/check*’ step involves the collection of data and analysis to determine if the changes made in the ‘Do/Change’ step are effective. This step occurs in union with the previous ‘Do/Change’ step as it involves the collection and evaluation of data regarding the effectiveness of the previous step. As such, measurements need to be enacted at a similar time to the previous step. Greene and colleagues [47] indicate the importance of collecting data and analysing results to determine what aspects of the changed implementation do and do not work. They state the importance of collecting feedback from everyone affected by the changes to determine a holistic picture [47].

Based on the results of the ‘Study/evaluate/check’ step, the ‘*Act*’ step required the stakeholder team to make a decision based on the data collected to either update *“act to hold”* [44], i.e. maintain the optimisation improvements they have made, or continue improvements. An example of a way to conduct this would be to formalise new policies or procedures, or hiring, reassigning or training staff [44].

The Plan-Do-Study-Act cycle is continuously conducted though some frameworks did reach a final sub-step. ‘*Sustain/endure*’ was a specific step dedicated to the maintenance of the improvement, to make sure the improvements are sustained, with a succession plan determined to control future processes. Provonost et al. [48] identifies this as an effort to include the improvement in other organisation wide quality improvement efforts, obtaining resources, and continuing measurements and feedback on outcomes. ‘*Disseminate/extend’* was a step dedicated to the deliberate dissemination of results. For example, this may include extending the improvement to other teams or sites [48] or sharing results of the process to improve care for others [47].

## Discussion

This scoping review using systematic search methods identified 20 frameworks that described concepts and steps to optimise health care interventions undertaken within public, medical or health service settings. This is two to four times the number of frameworks that were reported in the only other previously published reviews which discussed a) 10 purposively selected frameworks and models for implementation and improvement [23] and b) five frameworks for optimising intervention prior to conducting a randomised controlled trial [2]. This review significantly adds to the current evidence base on how to support the translation of evidence into practice. The identified frameworks on average included six steps where explicit guidance was available to support the conduct of optimisation processes. The majority of frameworks were cyclic or both cyclic and linear in nature, highlighting that certain aspects of optimisation are likely to be ongoing or iterative, potentially until a pre-specified outcome or endpoint is achieved.

Surprisingly, less than one third of the included frameworks specified an endpoint (i.e. where the process of optimisation ends). As optimisation processes may take considerable time and incur significant cost within typically resource-limited environments, some guidance on defining end-points or acceptable parameters to exit the optimisation cycle is likely to be useful for those seeking to optimise health interventions and/or their implementation. Such findings suggest that while some guidance exists to support the optimisation of public health interventions and their implementation, some refinements particularly with pre-specifying of end-points may improve the use of these frameworks in practice.

Frameworks also described applying optimisation concepts across various optimisation focus (intervention optimisation, implementation optimisation or both). The majority of frameworks were applied to optimise at least one outcome as defined by the Proctor framework [31], with more than half (55%) optimising intervention effectiveness. The predominant focus on optimising intervention effectiveness is unsurprising given that the main goal of public health interventions are to produce positive health outcomes for the population. However, a recent qualitative study examining optimisation of health care innovations in public health found that it was important for outcomes of the optimisation process to be determined by key stakeholders and end-users of the intervention to generate the greatest impact [13]. Previous studies have reported that stakeholders, typically agencies and organisations that fund the implementation or delivery of interventions, often consider many outcomes in addition to effectiveness, including cost, appropriateness to context and reach, when determining whether an intervention is suitable for translation or scaling up at a population level [13].

In our narrative synthesis, we described two synthesised frameworks for optimisation. These two frameworks include the most common steps used to optimise interventions and/or their implementation, providing a practical way of optimising intervention and/or implementation. Common to both frameworks is the problem identification step, with clear details of the problem or the aim of the optimisation process necessary. Pilot testing was similarly a sub-step in each meta-framework, with some frameworks suggesting an initial testing of intervention/implementation changes and the effectiveness prior to a larger scale evaluation. It is interesting to note that Plan-Do-Study-Act cycle were the primary driver for the optimisation of implementation optimisation. This is probably due to the influence of manufacturing, engineering or information technology processes on health, the field where optimisation can be said to have originated [49].

While such description may be useful to provide an overview of optimisation processes, further opportunities to study and/or validate such frameworks and their impact on improving outcomes exist. Further, these two meta-frameworks are standalone, able to be used individually for optimisation. Future research may look to investigate how these meta-frameworks may act together, or if they can be blended into one process for optimising interventions from conception to large-scale real-world implementation. Despite the opportunities to improve health outcomes, these frameworks also highlight the complexity and potential challenges with optimising health interventions and the likely variability in application of such steps depending on the context in which optimisation is occurring.

### Strengths and limitations

A limitation of the study was the difficulty in coding outcomes to be optimised according to Proctor et al. [31]. Outcomes listed in frameworks, although analogous to those classified by Proctor et al., were often not well described or used alternate terminology, and were consequently challenging to identify. Too, numerous frameworks did not explicitly state what they aimed to optimise, with many only considering outcomes in their worked examples. There were also cases where outcomes were not listed at all because there was a step within the framework for the improvement team to establish outcomes as part of the optimisation process.

A strength of the study included use of established and systematic scoping review methodology as outlined by Johanna Briggs Institute and the use of consensus process for the inclusion of steps in the meta-frameworks.

## Conclusions

This review provides a useful summary of the characteristics and steps to optimise a health care intervention or its implementation according to established optimisation frameworks. Further opportunities to investigate and validate such frameworks and their impact on improving a range of outcomes exist.

## Supplementary Information

**Additional file 1 Supplementary File 1.** Search terms.

**Additional file 2 Supplementary File 2.** Characteristics of individual included frameworks.

**Additional file 3 Supplementary File 3.** Synthesised Frameworks.

## Data Availability

The datasets used and/or analysed during the current study are available from the corresponding author on reasonable request.

## Abbreviations

CQI: Continuous quality improvement
ICSI: Institute for Clinical Systems Integration
MRC: Medical Research Council
MOST: Multiphase Optimisation Strategy
NPT: Normalisation Process Theory
PRIME: Process Modelling in Implementation Research
QI: Quality improvement
SMART: Sequential, Multiple Assignment, Randomised Trial
SD: Standard deviation

## References

[1] Masters R, Anwar E, Collins B (2017). Return on investment of public health interventions: a systematic review. J Epidemiol Community Health.

[2] Levati S, Campbell P, Frost R (2016). Optimisation of complex health interventions prior to a randomised controlled trial: a scoping review of strategies used. Pilot Feasibility Stud.

[3] Campbell NC, Murray E, Darbyshire J (2007). Designing and evaluating complex interventions to improve health care. BMJ.

[4] Grimshaw JM, Eccles MP, Lavis JN (2012). Knowledge translation of research findings. Implement Sci.

[5] McCrabb S, Lane C, Hall A (2019). Scaling-up evidence-based obesity interventions: a systematic review assessing intervention adaptations and effectiveness and quantifying the scale-up penalty. Obes Rev.

[6] Yoong SL, Wolfenden L, Clinton-McHarg T (2014). Exploring the pragmatic and explanatory study design on outcomes of systematic reviews of public health interventions: a case study on obesity prevention trials. J Public Health.

[7] Al-Abri R (2007). Managing change in healthcare. Oman Med J.

[8] Berndt ER, Cockburn IM, Grépin KA (2006). The impact of incremental innovation in biopharmaceuticals. PharmacoEcon.

[9] Ioannidis JP, Greenland S, Hlatky MA (2014). Increasing value and reducing waste in research design, conduct, and analysis. Lancet.

[10] Lynn J, Baily MA, Bottrell M (2007). The ethics of using quality improvement methods in health care. Ann Intern.

[11] Mason SE, Nicolay CR, Darzi A (2015). The use of lean and six sigma methodologies in surgery: a systematic review. Surgeon.

[12] Wolfenden L, Yoong SL, Williams CM (2017). Embedding researchers in health service organizations improves research translation and health service performance: the Australian hunter New England population health example. J Clin Epidemiol.

[13] Wolfenden L, Bolsewicz K, Grady A (2019). Optimisation: defining and exploring a concept to enhance the impact of public health initiatives. Health Res Policy Syst.

[14] McClure J, Derry H, Riggs K (2012). Questions about quitting (Q2): design and methods of a multiphase optimization strategy (MOST) randomized screening experiment for an online, motivational smoking cessation intervention. Contemp Clin Trials.

[15] McClure JB, Peterson D, Derry H (2014). Exploring the “active ingredients” of an online smoking intervention: a randomized factorial trial. Nicotine Tob Res.

[16] Reilly KL, Reeves P, Deeming S (2018). Economic analysis of three interventions of different intensity in improving school implementation of a government healthy canteen policy in Australia: costs, incremental and relative cost effectiveness. BMC Public Health.

[17] Powell AE, Rushmeer RK, Davies HT (2009). A systematic narrative review of quality improvement models in health care.

[18] Collins LM, Murphy SA, Nair VN (2005). A strategy for optimizing and evaluating behavioral interventions. Ann Behav Med.

[19] Medical Research Council (2000). A framework for development and evaluation of RCTs for complex interventions to improve health.

[20] Craig P, Dieppe P, Macintyre S (2008). Developing and evaluating complex interventions: the new Medical Research Council guidance. BMJ.

[21] Walker AE, Grimshaw J, Johnston M (2003). PRIME–PRocess modelling in ImpleMEntation research: selecting a theoretical basis for interventions to change clinical practice. BMC Health Serv Res.

[22] Murray E, Treweek S, Pope C (2010). Normalisation process theory: a framework for developing, evaluating and implementing complex interventions. BMC Med.

[23] Reed JE, Green S, Howe C (2019). Translating evidence in complex systems: a comparative review of implementation and improvement frameworks. Int J Qual Health Care.

[24] Peters MD, Godfrey CM, Khalil H (2015). Guidance for conducting systematic scoping reviews. Int J Evid Based Healthc.

[25] Moullin JC, Sabater-Hernandez D, Fernandez-Llimos F (2015). A systematic review of implementation frameworks of innovations in healthcare and resulting generic implementation framework. Health Res Policy Syst..

[26] Nilsen P (2015). Making sense of implementation theories, models and frameworks. Implement Sci.

[27] Hodder RK, Wolfenden L, Kamper SJ (2016). Developing implementation science to improve the translation of research to address low back pain: a critical review. Best Pract Res Clin Rheumatol.

[28] Kaplan HC, Brady PW, Dritz MC (2010). The influence of context on quality improvement success in health care: a systematic review of the literature. Milbank Q.

[29] Gardner K, Sibthorpe B, Chan M (2018). Implementation of continuous quality improvement in Aboriginal and Torres Strait islander primary health care in Australia: a scoping systematic review. BMC Health Serv Res.

[30] Veritas Health Innovation (2019). Covidence systematic review software. Melbourne, Australia.

[31] Proctor E, Silmere H, Raghavan R (2011). Outcomes for implementation research: conceptual distinctions, measurement challenges, and research agenda. Admin Pol Ment Health.

[32] Escoffery C, Lebow-Skelley E, Udelson H (2019). A scoping study of frameworks for adapting public health evidence-based interventions. Trans Behav Med.

[33] Antony J (2002). Design for six Sigma: a breakthrough business improvement strategy for achieving competitive advantage. Work Study.

[34] Bastian ND, Munoz D, Ventura M (2016). A mixed-methods research framework for healthcare process improvement. J Pediatr Nurs.

[35] ISIXSIGMA Six Sigma DMAIC Roadmap (2000). ISIXSIGMA.

[36] Czajkowski SM, Powell LH, Adler N (2015). From ideas to efficacy: the ORBIT model for developing behavioral treatments for chronic diseases. Health Psychol.

[37] Collins LM, Nahum-Shani I, Almirall D (2014). Optimization of behavioral dynamic treatment regimens based on the sequential, multiple assignment, randomized trial (SMART). Clin Trials.

[38] Institute for Healthcare Improvement (2003). The breakthrough series IHI’s collaborative model for achieving breakthrough improvement.

[39] Abdelmotleb FA (2008). Development of Total quality management framework for Libyan health care organisations.

[40] Haji FA, Da Silva C, Daigle DT (2014). From bricks to buildings: adapting the medical research council framework to develop programs of research in simulation education and training for the health professions. Simul Healthc.

[41] McGonigal M (2017). Implementing a 4C approach to quality improvement. Crit Care Nurs Q.

[42] Sutton LJ, Jarden RJ (2017). Improving the quality of nurse-influenced patient care in the intensive care unit. Nurs Crit Care.

[43] McKay R, Coombs T, Pirkis J. A framework for exploring the potential of routine outcome measurement to improve mental health care. Australas Psychiatry 2012;20(2):127–133. 10.1177/1039856212436621. 10.1177/1039856212436621 22467560

[44] Redick EL (1999). Applying FOCUS-PDCA to solve clinical problems. Dimens Crit Care Nurs.

[45] Mosser G (1996). Clinical process improvement: engage first, measure later. Qual Manag Health Care.

[46] Taylor MJ, McNicholas C, Nicolay C (2014). Systematic review of the application of the plan–do–study–act method to improve quality in healthcare. BMJ Qual Saf.

[47] Greene SM, Reid RJ, Larson EB (2012). Implementing the learning health system: from concept to action. Ann Intern Med.

[48] Pronovost PJ, Berenholtz SM, Needham DM (2008). Translating evidence into practice: a model for large scale knowledge translation. BMJ.

[49] Moen R (2009). Foundation and History of the PDSA Cycle. Asian network for quality conference Tokyo.

