# Supplementary material for: How to optimise public health interventions: a scoping review of guidance from optimisation process frameworks
Source: BMC Public Health. 2020 Dec 2;20:1849. doi: 10.1186/s12889-020-09950-5 (PMC7709329; doi:10.1186/s12889-020-09950-5)
Supplement: Supplementary file 1 — Additional file 1 Supplementary File 1. Search terms. [file 12889_2020_9950_MOESM1_ESM.docx]

**Supplementary File 1- Search terms**

**Table 1.** Search terms for the first round of searching^*^

| **Improvement terms (Optimisation, QI, and CQI)** | **1** | (“quality improvement*” or “improve* quality” or “quality management*” or “improve* patient care” or “process improvement”).ti,ab. |
| --- | --- | --- |
|  | **2** | “Continuous Quality Improvement”.ti,ab. |
|  | **3** | “Total quality management”.ti,ab. |
|  | **4** | (“optimisation” or “optimization” or “optimi*”).ti,ab. |
|  | **5** | Or/1-4 |
| **Context** | **6** | (community or community-based or clinical health or health care or healthcare or primary health or health service$ or medical or public health).ti,ab. |
| **Frameworks** | **7** | (framework* adj5 improv*).ti,ab |
|  | **8** | (framework* adj5 impact).ti,ab. |
|  | **9** | (framework* adj5 implem*).ti,ab. |
|  | **10** | (framework* adj5 accept*).ti,ab. |
|  | **11** | (framework* adj5 effect*).ti,ab. |
|  | **12** | (framework* adj5 qualit*).ti,ab. |
|  | **13** | (framework* adj5 optim*).ti,ab. |
|  | **14** | (framework* adj5 scal*).ti,ab. |
|  | **15** | (framework* adj5 transla*).ti,ab. |
|  | **16** | Or/7-15 |
| **All** | **17** | 5 and 6 and 16 |
| **Limits** | **18** | Limit 17 to English year 2003-current |

^*^ A lack of chronic health specific articles were identified in the first search. Additional criteria were added to the second search to ensure they were captured by the search strategy.

**Table 2.** Search terms for the second search (additional terms are in bold).

| **Improvement terms (Optimisation, QI, and CQI)** | **1** | (“quality improvement*” or “improve* quality” or “quality management*” or “improve* patient care” or “process improvement”).ti,ab. |
| --- | --- | --- |
|  | **2** | “Continuous Quality Improvement”.ti,ab. |
|  | **3** | “Total quality management”.ti,ab. |
|  | **4** | (“optimisation” or “optimization” or “optimi*”).ti,ab. |
|  | **5** | Or/1-4 |
| **Context** | **6** | (community or community-based or clinical health or health care or healthcare or primary health or health service$ or medical or public health **or chronic disease or smok* or tobacco or nutrition or diet or obesity or alcohol or physical activity or disease**).ti,ab. |
| **Frameworks** | **7** | (framework* adj5 improv*).ti,ab |
|  | **8** | (framework* adj5 impact).ti,ab. |
|  | **9** | (framework* adj5 implem*).ti,ab. |
|  | **10** | (framework* adj5 accept*).ti,ab. |
|  | **11** | (framework* adj5 effect*).ti,ab. |
|  | **12** | (framework* adj5 qualit*).ti,ab. |
|  | **13** | (framework* adj5 optim*).ti,ab. |
|  | **14** | (framework* adj5 scal*).ti,ab. |
|  | **15** | (framework* adj5 transla*).ti,ab. |
|  | **16** | **(framework* adj5 develop*).ti,ab.** |
|  | **17** | **(framework* adj5 intervention).ti,ab.** |
|  | **18** | Or/7-17 |
| **All** | **19** | 5 and 6 and 18 |
| **Limits** | **20** | Limit 19 to English year 2003-current |
