# Supplementary material for: How to optimise public health interventions: a scoping review of guidance from optimisation process frameworks
Source: BMC Public Health. 2020 Dec 2;20:1849. doi: 10.1186/s12889-020-09950-5 (PMC7709329; doi:10.1186/s12889-020-09950-5)
Supplement: Supplementary file 2 — Additional file 2 Supplementary File 2. Characteristics of individual included frameworks. [file 12889_2020_9950_MOESM2_ESM.docx]

**Supplementary File 2.** Characteristics of individual included frameworks.

| **Author, year** | **Framework Name** | **Number of steps** | **Figure available** | **Explicit guidance for each step available** | **Framework format** | **Endpoint specified** | **Optimisation focus** | **Modified from other frameworks** |
| --- | --- | --- | --- | --- | --- | --- | --- | --- |
| Abdelmotleb et al., 2008^1^ | The proposed TQM framework for Libyan healthcare sector | 8 | Yes | Yes | Linear and cyclic |  | Both intervention and implementation | Yes |
| Antony et al., 2002^2^ | Design for Six Sigma (DFSS) | 4 | Yes | Yes | Linear and cyclic | Yes | Implementation |  |
| Bastian et al., 2016^3^ | Mixed-Methods Research Framework | 4 | Yes | Yes | Linear | Yes | Implementation | Yes |
| Collins et al., 2005^4^ | Multiphase optimization strategy (MOST) for fixed treatments | 9 |  | Yes | Linear | Yes | Intervention |  |
| Collins et al., 2014^5^ | The multiphase optimization strategy (MOST) for dynamic treatment regimes | 3 |  | Yes | Cyclic |  | Intervention |  |
| Craig et al., 2008^6^ | Medical Research Council (MRC) 2008 | 4 | Yes | Yes | Cyclic |  | Intervention | Yes |
| Czajkowski et al., 2015^7^ | ORBIT Model (Obesity-Related Behavioral Intervention Trials) | 5 | Yes | Yes | Linear and cyclic | Yes | Intervention |  |
| Greene et al., 2012^8^ | Rapid learning health care system | 6 | Yes | Yes | Cyclic |  | Implementation |  |
| Haji et al., 2014^9^ | Modified MRC's framework | 8 | Yes | Yes | Cyclic |  | Intervention | Yes |
| Institute for Healthcare Improvement et al., 2003^10^ | Model for Improvement | 7 | Yes |  | Linear and cyclic | Yes | Implementation |  |
| ISIXSIGMA et al., 2019^11^ | Six sigma DMAIC (Define, Measure, Analyze, Improve, Control) | 5 |  | Yes | Linear and cyclic | Yes | Implementation |  |
| McGonigal et al., 2017^12^ | 4C model | 4 | Yes | Yes | Cyclic |  | Implementation | Yes |
| McKay et al., 2012^13^ | Utilisation of Routine Outcome Measurement | 5 | Yes | Yes | Cyclic |  | Implementation |  |
| Medical Research Council et al., 2000^14^ | Medical Research Council's (MRCs) framework | 5 | Yes | Yes | Linear and cyclic |  | Intervention |  |
| Mosser et al., 1996^15^ | ICSI process specification for the prevention or treatment of a given diagnosis guideline | 9 | Yes | Yes | Linear |  | Implementation |  |
| Murray et al., 2010^16^ | Normalisation Process Theory | 5 |  | Yes | Linear and cyclic |  | Intervention |  |
| Pronovost et al., 2008^17^ | Johns Hopkins Quality and Safety Research Group translating evidence into practice model | 9 | Yes | Yes | Linear and cyclic |  | Implementation |  |
| Redick et al., 1999^18^ | FOCUS-PDCA | 9 |  | Yes | Linear |  | Implementation |  |
| Sutton et al., 2016^19^ | Developed their own framework based on Eight steps, Constructive feedback (DEARMAN), PDSA | 4 | Yes | Yes | Cyclic |  | Implementation | Yes |
| Zhou et al., 2019^20^ | EPIDEM | 6 |  | Yes | Cyclic |  | Intervention |  |

**References**

1. Abdelmotleb FA. Development of Total Quality Management framework for Libyan health care organisations. UK: Sheffield Hallam University; 2008.

2. Antony J. Design for Six Sigma: a breakthrough business improvement strategy for achieving competitive advantage. Work Study. 2002 Feb 1;51(1):6-8. https://doi.org/10.1108/00438020210415460

3. Bastian ND, Munoz D, Ventura M. A mixed-methods research framework for healthcare process improvement. J Pediatr Nurs. 2016 Jan 1;31(1):e39-e51. https://doi.org/10.1016/j.pedn.2015.09.003

4. Collins LM, Murphy SA, Nair VN, et al. A strategy for optimizing and evaluating behavioral interventions. Ann Behav Med. 2005 Feb 1;30(1):65-73. https://doi.org/10.1207/s15324796abm3001_8

5. Collins LM, Nahum-Shani I, Almirall D. Optimization of behavioral dynamic treatment regimens based on the sequential, multiple assignment, randomized trial (SMART). Clin Trials. 2014 Aug;11(4):426-34. https://doi.org/10.1177/1740774514536795

6. Craig P, Dieppe P, Macintyre S, et al. Developing and evaluating complex interventions: the new Medical Research Council guidance. BMJ. 2008 Sep 29;337. https://doi.org/10.1136/bmj.a1655

7. Czajkowski SM, Powell LH, Adler N, et al. From ideas to efficacy: The ORBIT model for developing behavioral treatments for chronic diseases. Health Psychol. 2015 Oct;34(10):971. https://doi.org/10.1037/hea0000161

8. Greene SM, Reid RJ, Larson EB. Implementing the learning health system: from concept to action. Ann Intern Med. 2012 Aug 7;157(3):207-10. doi:10.7326/0003-4819-157-3-201208070-00012

9. Haji FA, Da Silva C, Daigle DT, et al. From bricks to buildings: adapting the medical research council framework to develop programs of research in simulation education and training for the health professions. Simul Healthc. 2014 Aug 1;9(4):249-59. doi:10.1097/SIH.0000000000000039

10. Institute for Healthcare Improvement. The Breakthrough Series IHI’s Collaborative Model for Achieving Breakthrough Improvement. Cambridge: IHI; 2003.

11. ISIXSIGMA Six Sigma DMAIC Roadmap: ISIXSIGMA. https://www.isixsigma.com/new-to-six-sigma/dmaic/six-sigma-dmaic-roadmap/ (2000). Accessed 12 Jan 2019.

12. McGonigal M. Implementing a 4C Approach to Quality Improvement. Crit Care Nurs Q. 2017 Jan 1;40(1):3-7. https://doi.org/10.1097/CNQ.0000000000000134

13. McKay R, Coombs T, Pirkis J. A framework for exploring the potential of routine outcome measurement to improve mental health care. Australas Psychiatry. 2012 Apr;20(2):127-33. https://doi.org/10.1177/1039856212436621

14. Medical Research Council. A framework for development and evaluation of RCTs for complex interventions to improve health. UK: Medical Research Council; 2000.

15. Mosser G. Clinical process improvement: engage first, measure later. Qual Manag Health Care. 1996;4(4):11-20. doi:10.1097/00019514-199604040-00003

16. Murray E, Treweek S, Pope C, et al. Normalisation process theory: a framework for developing, evaluating and implementing complex interventions. BMC Med. 2010 Dec 1;8(1):63. https://doi.org/10.1186/1741-7015-8-63

17. Pronovost PJ, Berenholtz SM, Needham DM. Translating evidence into practice: a model for large scale knowledge translation. BMJ. 2008 Oct 6;337:a1714. https://doi.org/10.1136/bmj.a1714

18. Redick EL. Applying FOCUS-PDCA to solve clinical problems. Dimens Crit Care Nurs. 1999 Nov 1;18(6):30.

19. Sutton LJ, Jarden RJ. Improving the quality of nurse‐influenced patient care in the intensive care unit. Nurs Crit Care. 2017 Nov;22(6):339-47. https://doi.org/10.1111/nicc.12266

20. Zhou Y. EPIDEM: A Model for Quality Improvement. Lab Med. 2019 Jan 1;50(1):e9-14.
