# Supplementary material for: How to optimise public health interventions: a scoping review of guidance from optimisation process frameworks
Source: BMC Public Health. 2020 Dec 2;20:1849. doi: 10.1186/s12889-020-09950-5 (PMC7709329; doi:10.1186/s12889-020-09950-5)
Supplement: Supplementary file 3 — Additional file 3 Supplementary File 3. Synthesised Frameworks. [file 12889_2020_9950_MOESM3_ESM.docx]

**Supplementary File 3- Synthesised Frameworks**

**Table 1.** Intervention optimisation

| **Step** | **Information from framework** |
| --- | --- |
| Problem identification | - Identify purpose of the research and primary research questions^1^  - Identify the problem in the current quality system^2^  - Significant clinical question^3^  - Clarify whether a problem exists, why there is a problem, the scope of the problem, and any potential solutions.^4^ |
| Preparation | - Form theoretical model based on data, experience, and literature. Develop and pilot intervention. Define optimisation criteria^5^  - Screening^6^  - Define the intervention^7^ |
| *Theoretical/Literature base* | *- Define and refine basic logic and elements of intervention ^3^*  *- Theory and modelling to identify components and mechanisms^1, 8, 9^* |
| *Pilot/Feasibility testing* | *- Proof of concept pilot testing^3^*  *- Pilot and assess feasibility^9^*  - Conduct NPT analysis of trail parameters to determine effectiveness^7^ |
| Optimisation | - experimentation and secondary data analysis to ensure effect (of components) against optimisation criteria^5^  - Refine^6^  - Describe constant and variable components of replicable intervention^8^  - What is the optimal design of the intervention? Is it acceptable/feasible?^1^ |
| Evaluation | - Does optimised intervention produce clinical significant benefits^5^  - Compare fully defined intervention to appropriate alternative^1, 8^  - Assess effectiveness and intervention processes (e.g. implementation) to clarify casual mechanisms and contextual factors^9^  - Evaluate the project’s success using meaningful measures.^4^ |
| Long-term implementation | - Determine whether intervention can replicate the intervention in uncontrolled settings long-term^1, 8, 9^  - Once an intervention has been proven effective, the next step is to ensure wide scale implementation^7^ |

*Italics indicates sub-steps*

**Figure 1.** Intervention optimisation

**Preparation**

**Problem Identification**

**Evaluation**

*Theoretical/literature base*

*Pilot/feasibility testing*

**Optimisation**

**Long-term implementation**

**Table 2.** Implementation optimisation

| **Stage** | **Example from frameworks** |
| --- | --- |
| Problem identification | - Centre on the issue at hand^10^  - Identify problem & criteria for screens.^11^  - Use criteria in selecting topics- free of condition, probability and improvement, anticipated magnitude of improvement^12^  - Use data to inform choices^13^  - Envision the problem within a larger healthcare system^14^  - Define the project goal, customer & deliverables, quantify the problem with data^15^  - Find a process to improve- is the problem high volume, high cost, high risk or high interest, is the study feasible, measureable, significant^16^  - Determine what you’re trying to accomplish^17^  - Understand service setting, mode of operation and culture and available resources. Prioritise areas for improvement, determine the practice to be improved and decide outcomes and how they will be assessed^18^ |
| Collaborate | - Involve the team members/those impacted by the change^10^  - identify and engage key personnel & build a local QI team^19^  - Form guideline working group that is multidisciplinary depending on topic^12^  - Involve key stakeholders^13^  - Engage collaborative multidisciplinary teams^14^  - Enlist stakeholders to share concerns and identify problems/barriers/ potential gains^14^  - Organise a team that includes representation for all disciplines involved in the process ^16^ |
| Plan/design | - Identify local reporting systems for development of initiative^19^  - Literature reviews to identify standards^19^  - Analysis of design requirements and parameters^11^  - Design case and evaluation based on local and external evidence^13^  - Measure baseline performance; determine root cause^15^  - Clarify current knowledge, understand cause of variation, planed improvement^16^  - Conduct literature search and stake holder analysis to understand issues. Time motion study/root cause analysis etc. ^20^  - Identify changes that will result in improvement^17^  - Ensure that relevant evidence that guide action, identify practices that require modification^18^ |
| *Pilot* | *- Develop and pilot test measures, measure baseline performance^14^*  *- Develop measures^20^*  *- Pilot^12, 18^* |
| Do/change | - Communicate vision, create quick wins, implement the QI strategy ^19^  - Install/re-install guidelines^12^  - Apply the plan in pilot and control settings^13^  - Design a toolkit targeted at barriers, independent checks, reminders and learning from mistakes. Explain importance of intervention and share evidence supporting the intervention^14^  - Improve the process by eliminating defects^15^  - Implement the plan^16, 17^  - Inform related changes in practice^18^ |
| Study/evaluate/check | - Establish data collection tools and methods^19^  - Identify sources of variability, tolerance, reliability. Check product meets criteria ^11^  - Measure, receive and analyse data^12^  - Collect data and analyse results to show what does and does not work and facilitate adoption system wide^13^  - Assess performance measures and unintended consequences^14^  - Check data for customer improvement and process improvement^16, 17^  - Review achievements and challenges at set points^18^ |
| Act | - Revise guidelines, use data and additional literature^12^  - Adjust and use evidence for continual improvement^13^  - Act to hold, gain or continue improvement e.g. formalise new policies and procedures^16, 17^ |
| *Sustain/endure* | *- Maintain urgency with reporting, improve systems and processes, succession planning^19^*  *- Endure: integrate into quality improvement initiatives, audit and feedback, resources, spread the intervention into other departments ^14^*  *- Control future process performance^15^* |
| *Disseminate/extend* | *- Shore results to improve core for everyone. Use deliberate and timely internal dissemination ^13^*  *- Extend^14^* |

*Italics indicates sub-steps*

**Figure 2.** Implementation optimisation

**Study/evaluate/check**

**Collaborate**

**Problem identification**

**Plan/design**

**Optimisation phase**

*Disseminate/extend*

*Sustain/endure*

**Act**

**Do/change**

*Pilot*

**References**

1. Haji FA, Da Silva C, Daigle DT, et al. From bricks to buildings: adapting the medical research council framework to develop programs of research in simulation education and training for the health professions. Simul Healthc. 2014 Aug 1;9(4):249-59. doi:10.1097/SIH.0000000000000039.

2. Abdelmotleb FA. Development of Total Quality Management framework for Libyan health care organisations. UK: Sheffield Hallam University; 2008.

3. Czajkowski SM, Powell LH, Adler N, et al. From ideas to efficacy: The ORBIT model for developing behavioral treatments for chronic diseases. Health Psychol. 2015 Oct;34(10):971. https://doi.org/10.1037/hea0000161.

4. Zhou Y. EPIDEM: A Model for Quality Improvement. Lab Med. 2019 Jan 1;50(1):e9-14. doi:10.1093/labmed/lmy066.

5. Collins LM, Nahum-Shani I, Almirall D. Optimization of behavioral dynamic treatment regimens based on the sequential, multiple assignment, randomized trial (SMART). Clin Trials. 2014 Aug;11(4):426-34. doi:10.1177/1740774514536795.

6. Collins LM, Murphy SA, Nair VN, et al. A strategy for optimizing and evaluating behavioral interventions. Ann Behav Med. 2005 Feb 1;30(1):65-73. doi:10.1207/s15324796abm3001_8.

7. Murray E, Treweek S, Pope C, et al. Normalisation process theory: a framework for developing, evaluating and implementing complex interventions. BMC Med. 2010 Dec 1;8(1):63. doi:10.1186/1741-7015-8-63.

8. Medical Research Council. A framework for development and evaluation of RCTs for complex interventions to improve health. UK: Medical Research Council; 2000.

9. Craig P, Dieppe P, Macintyre S, et al. Developing and evaluating complex interventions: the new Medical Research Council guidance. BMJ. 2008;337:a1655. doi:10.1136/bmj.a1655.

10. McGonigal M. Implementing a 4C Approach to Quality Improvement. Crit Care Nurs Q. 2017 Jan 1;40(1):3-7. https://doi.org/10.1097/CNQ.0000000000000134.

11. Antony J. Design for Six Sigma: a breakthrough business improvement strategy for achieving competitive advantage. Work Study. 2002 Feb 1;51(1):6-8. https://doi.org/10.1108/00438020210415460.

12. Mosser G. Clinical process improvement: engage first, measure later. Qual Manag Health Care. 1996;4(4):11-20. doi:10.1097/00019514-199604040-00003.

13. Greene SM, Reid RJ, Larson EB. Implementing the learning health system: from concept to action. Ann Intern Med. 2012 Aug 7;157(3):207-10. doi:10.7326/0003-4819-157-3-201208070-00012.

14. Pronovost PJ, Berenholtz SM, Needham DM. Translating evidence into practice: a model for large scale knowledge translation. BMJ. 2008 Oct 6;337:a1714. https://doi.org/10.1136/bmj.a1714.

15. ISIXSIGMA Six Sigma DMAIC Roadmap: ISIXSIGMA. https://www.isixsigma.com/new-to-six-sigma/dmaic/six-sigma-dmaic-roadmap/ (2000). Accessed 12 Jan 2019.

16. Redick EL. Applying FOCUS-PDCA to solve clinical problems. Dimens Crit Care Nurs. 1999 Nov 1;18(6):30.

17. Institute for Healthcare Improvement. The Breakthrough Series IHI’s Collaborative Model for Achieving Breakthrough Improvement. Cambridge: IHI; 2003.

18. McKay R, Coombs T, Pirkis J. A framework for exploring the potential of routine outcome measurement to improve mental health care. Australas Psychiatry. 2012 Apr;20(2):127-33. https://doi.org/10.1177/1039856212436621

19. Sutton LJ, Jarden RJ. Improving the quality of nurse‐influenced patient care in the intensive care unit. Nurs Crit Care. 2017 Nov;22(6):339-47. https://doi.org/10.1111/nicc.12266

20. Bastian ND, Munoz D, Ventura M. A mixed-methods research framework for healthcare process improvement. J Pediatr Nursing. 2016 Jan 1;31(1):e39-e51. doi:10.1016/j.pedn.2015.09.003
